# *In Vivo* Efficacy of Artesunate/Sulphadoxine-Pyrimethamine *versus* Artesunate/Amodiaquine in the Treatment of Uncomplicated *P. falciparium* Malaria in Children around the Slope of Mount Cameroon: A Randomized Controlled Trial

**DOI:** 10.3390/biomedicines4010005

**Published:** 2016-02-15

**Authors:** Tobias O. Apinjoh, Judith K. Anchang-Kimbi, Marcelus U. Ajonina, Esther T. Njonguo, Clarisse Njua-Yafi, Andre N. Ngwai, Regina N. Mugri, Eric A. Achidi

**Affiliations:** 1 Department of Biochemistry and Molecular Biology, University of Buea, PO Box 63 Buea, Cameroon; apinjohtoby@yahoo.co.uk (T.O.A.); majonin@gmail.com (E.T.N.); 2 Department of Zoology and Animal Physiology, University of Buea, PO Box 63 Buea, Cameroon; kuoh2000@yahoo.fr; 3 School of Applied Sciences, Saint Monica University, PO Box 132 Buea, Cameroon; majonina@stmonicauniversity.com; 4 Department of Animal Biology and Physiology, University of Yaounde I, PO Box 8094 Yaounde, Cameroon; ncyafi2000@yahoo.com; 5 Department of Medical Laboratory Science, University of Buea, PO Box 63 Buea, Cameroon; ndingwai2000@yahoo.com (A.N.N.); mugri72@yahoo.com (R.N.M.)

**Keywords:** *in vivo* efficacy, ACT (artemisinin–based combination therapy), uncomplicated *Plasmodium falciparum*

## Abstract

Background: The development and spread of antimalarial drug resistant parasites contributes to the global impact of the disease. *In vivo* efficacy assessments of treatments for *Plasmodium falciparum* malaria are essential for ensuring effective case management. Artemisinin-based combinations have been adopted as the first-line treatment for uncomplicated *P. falciparum* malaria in Cameroon since 2004. Methods: A total of 177 children aged six-months to 10 years with uncomplicated mono-infected falciparum malaria were randomized (1:1) to receive artesunate/sulphadoxine-pyrimethamine (AS/SP) or artesunate/amodiaquine (AS/AQ) pediatric tablets and followed up for 28 days according to the standard World Health Organization *in vivo* drug efficacy monitoring protocol. The primary and secondary endpoints were PCR uncorrected and corrected cure rates, as measured by adequate clinical and parasitological response (ACPR) on day 28. Results: The PCR corrected cure rate was high, overall (88.1%, 95% CI 83.1–93.1), 85.9% (95% CI 78.2–93.6), and 90.2% (95% CI 83.8–96.6) for AS/SP and AS/AQ, respectively. Twenty-one treatment failures were observed during follow-up, constituting one (4.6%), 14 (8.2%), and six (3.5%) early treatment failure (ETF), late clinical failure (LCF), and late parasitological failure (LPF), respectively. The drugs were well tolerated with no serious adverse events. Conclusions: Both AS/SP and AS/AQ are highly effective and well-tolerated treatments for uncomplicated *P. falciparum* malaria around the slope of Mount Cameroon.

## 1. Introduction

Malaria due to *Plasmodium falciparum* is the leading cause of morbidity and mortality in Africa, especially in children under the age of five years, where a child dies every minute [1]. The disease remains a major public health concern in spite of the scale up in control interventions [2,3] due partly to the development and spread of antimalarial drug resistant parasites [4]. Resistance to traditional antimalarials, particularly chloroquine (CQ), the mainstay of 20th century malaria eradication [4] and sulfadoxine-pyrimethamine (SP), the only alternative available for large scale implementation [2] led to sweeping changes in antimalarial treatment recommendations. These therapies have been compromised by the spread of drug resistance leading to the World Health Organization (WHO) recommending the use of Artemisinin-based combination therapies (ACTs) as the first-line treatment regimen for uncomplicated malaria in all endemic regions [5]. ACTs combine an artemisinin-derivative, usually dihydroartemisinin, artesunate or artemether, with another longer-lasting drug such as lumefantrine, mefloquine, amodiaquine, sulfadoxine/pyrimethamine, and piperaquine, with different mode of action to try and reduce the risk of further resistance developing and to lower the treatment course [6]. The artemisinin derivatives are effective not only against multi-resistant strains of *P. falciparum*, but have broad stage specificity against the *Plasmodium* life cycle including activity throughout the asexual blood and sexual gametocyte stages [7,8]. Artemisinin derivatives have been shown to produce faster relief of clinical symptoms and faster clearance of parasites from the blood compared to other antimalarial drugs [2,9,10]. WHO recommends five ACTs for the treatment of uncomplicated malaria in endemic regions: dihydroartemisinin-piperaquine (DHA-P), artesunate-mefloquine (AS + MQ), artemether-lumefantrine (AL), artesunate-amodiaquine (AS + AQ), and artesunate plus sulfadoxine-pyrimethamine (AS + SP) [5].

Although ACTs remains the most effective drug for the treatment of malaria, a declining efficacy of this regime has been reported in some malaria-endemic regions [11]. A resistant phenotype has been detected in five countries of the Greater Mekong Sub-region: Cambodia, the Lao People’s Democratic Republic, Myanmar, Thailand, and Vietnam, as relatively slow parasite clearance rates in patients receiving artemisinin or ACT [12,13,14]. This, therefore, suggests that resistant parasite strains have emerged, prompting fears that they may have spread over to other endemic regions, especially in Africa, as was the case with chloroquine [15]. A recent study in Angola revealed a treatment failure after oral treatment with dihydroartemisinin/piperaquine, suggesting that resistant strains may already have spread to Africa [16]. As such, the rapid detection of new artemisinin resistance foci and implementation of containment interventions are urgently needed to prevent the spread of resistant parasites [1].

In order to detect early changes to *P. falciparium* sensitivity to ACTs, WHO recommends the monitoring of their therapeutic efficacy every two years in all endemic countries especially in countries of known resistance to partner drugs [17]. *In vivo* efficacy studies and the use of molecular markers are important tools employed in the early detection of drug resistance [18,19,20]. *In vivo* assays involve direct measurement of parasite drug responses on patients or indirectly measuring the prevalence of resistance-associated mutations within a parasite population. These assays allow the components of combination therapies to be tested individually against parasites, and they can detect decreases in drug efficacy before resistance becomes clinically evident and widespread [21]. Parasite clearance rate (PCT) and its related clinical phenotype (delayed PCT) are currently the best practical surrogates of artemisinin *in vivo* resistance [22]. However, the frequent sampling (every six or eight hours) required to measure PCT accurately and to estimate the parasite clearance half-life [23] makes it practically difficult even in research settings. Alternatively, treatment failure can quite simply and accurately be predicted as a failure to clear parasites by Day 3 (72 h post-treatment start) [24], requiring a single time-point and, thus, limiting the workload in resource-limited settings.

In Cameroon, chloroquine was replaced by AQ and SP by the beginning of the 21st century due to the emergence and spread of resistant strains to the drug across the country [25,26,27]. Moreover, a decrease in sensitivity of *P. falciparum* to AQ and SP in the country led to adoption of ACTs as the first line treatment for uncomplicated *P. falciparum* by the Ministry of Public Health since 2004 [28], with Artesunate-amodiaquine (AS/AQ) and artemether-lumefantrine (AL) currently recommended [29]. Although there have been previous reports on the efficacies of the AS/AQ combination and treatment failures in the center and northern regions of the country [28,30,31], studies in the southwestern region have been limited. There is therefore a need for routine monitoring of the efficacy of these antimalarials to identify and contain the spread of *P. falciparum*-resistant strains in regions where the disease is endemic. The aim of this study was to determine and compare the efficacies of two antimalarial combination regimens (AS/SP and AS/AQ) for the treatment of uncomplicated *P. falciparum* malaria in children at the slope of Mount Cameroon.

## 2. Experimental Section

### 2.1. Study Area

The study was carried out between May and October 2006 in the Buea Regional Hospital (BRH) and Tole-Bwyuiku Health Post (THP), along the eastern slope of Mount Cameroon. While BRH is the main government institution in the town, THP was the only functional health facility in the village at the time. The area has a forested equatorial climate, modified by the ocean and mountain and has two seasons: a short dry season (November–March) and a long rainy season (March–November) [32,33]. It is characterized by fairly constant temperatures that vary from 18 °C in August to 35 °C in March. The relative humidity (75%–80%), average annual rainfall (2625 mm) and precipitation (2000–10,000 mm) are relatively high [32]. Malaria transmission is intense and perennial in the area, with parasitemia higher in the rainy seasons and at lower altitude [33]. *Plasmodium falciparum* accounts for most of the infections while *Anopheles gambiae* is the dominant, most aggressive, and most active of the three malaria vectors in the area (*An. gambiae*, *An. Funestus*, and *An. nili*) [32].

### 2.2. Study Design

This study compared the therapeutic efficacy of artesunate plus amodiaquine (AS/AQ) and artesunate plus sulphadoxine/pyrimethamine (AS/SP) for uncomplicated *P. falciparum* mono-infection, based on clinical and parasitological parameters through a prospective, 28-day, open label, *in vivo* efficacy assessment according to the 2003 WHO protocol for therapeutic efficacy [2,34].

### 2.3. Sample Size Calculation

*A priori* power calculation was used to determine the sample size required to demonstrate no difference in proportion of adequate clinical responses between the AS/SP and the AS/AQ groups, assuming an intergroup difference <10% in the target population of children under five years of age, a type I error of 5% and a power of 80%. A minimum of 65 participants was, thus, required to obtain meaningful and cost effective results. Assuming a loss to follow-up rate estimated of 20% gave a total of 168 patients; at least 82 per treatment group.

### 2.4. Patients

Patients aged 6–72 months with *P. falciparum* mono infection, and parasitemia levels of at least 1000 asexual forms per µL were enrolled. Additional inclusion criteria included axillary temperature ≥37.5 °C or history of fever within the past 24 h and residence within 20 km of the study site to facilitate follow-up. Children were excluded if they presented with the following: detection of any Plasmodium infection besides falciparum, signs or symptoms of severe malaria, concomitant febrile illness, history of allergy to study drugs or known allergy to other antimalarial drugs, evidence of underlying chronic diseases (cardiac, renal, hepatic, and malnutrition). Other exclusion criteria included residence out of the study area, parents/guardian’s unwillingness to provide written informed assent, and inability to take oral medication. Children were withdrawn from the study if any of the following occurred: (1) use of antimalarial drugs outside of the study protocol; (2) concomitant febrile illness; (3) withdrawal of assent; (4) protocol violation; and (5) loss to follow-up [34].

### 2.5. Clinical and Laboratory Procedures

All sick children attending the outpatient ward of BRH or THP were initially evaluated by the clinical staff and those with fever or a history of fever were referred for malaria testing. Thick and thin blood smears were prepared from all patients at initial presentation at the health facility, following standard procedures and stained with 10% Giemsa (Sigma, St. Louis, MO, USA). The malaria status and parasite density were determined under oil immersion with the 100× objective, 10× eyepiece of a binocular microscope (Olympus Optical Co., Ltd, Tokyo, Japan) while the *Plasmodium* species were identified on the thin blood smear. A smear was only considered negative if no malaria parasites were seen in 100 high-power fields. With each positive smear, the level of parasitemia was estimated by counting the parasites against at least 200 leucocytes and assuming a leucocyte count of 8000 per microliter to calculate the number of parasites/µL blood [33]. All blood films were read by two independent microscopists and slides with parasite densities differing by more than 20% between microscopists were reassessed by a third microscopist, with the third reading considered final [35]. After meeting the inclusion criteria and consenting to enrollment into the study, all patients underwent hemoglobin (Hb) testing (Hemocue, Angelholm, Sweden) and filter paper blood spot collection. Anemia was defined as Hb < 11 g/dL and further classified as severe, moderate, or mild as reported previously [33].

### 2.6. Treatment

Following the parents/guardians’ interview and child’s examination by the study clinician, patients were allocated to either of the two treatment groups by sequential alteration. Children were given an oral antimalarial based on weight according to national guidelines as follows: (1) 4 mg/kg/day artesunate for three days plus 15 mg/kg sulphadoxine/pyrimethamine as a single dose and (2) 4 mg/kg/day artesunate plus 10 mg/kg/day amodiaquine packaged in fixed-dose combination tablets for three days. Parents/guardians were instructed to administer the evening dose with food. On follow-up day 3, parents/guardians were asked if the drug was given properly the previous two days. Patients that did not return on schedule for follow-up were visited at home on the same day.

The initial and each morning dose were directly observed by the study staff milk and biscuits were given alongside the drugs to facilitate drug absorption [36]. Patients were monitored by the study nurse or pharmacist for 30 min and a full dose re-administered if the child vomited the drug within 30 min. If the patient vomited again, he or she was referred to the hospital and withdrawn from the study. An antipyretic (15 mg/kg paracetamol every eight hours, for 24 h) was given on days 0, 1, and 2 for temperatures >38 °C, as per national guidelines [36]. Moreover, ferrous sulfate and folate were given to all children with hemoglobin <10 g/dL as per Integrated Management of Childhood Illness (IMCI) guidelines [37]. Quinine (10 mg/kg every 8 h for seven days), the second-line treatment as per national policy, was administered as rescue therapy [36].

### 2.7. Follow-Up

Patients were followed-up for 28 days and asked to return on days 3, 7, 14, and 28 post-ACT treatment initiation, as well as any other interim day, if ill. The study site facilities were open from 7:30 am to 5:00 pm and after these hours care was also available. Standardized follow-up included documentation of physical examination including axillary temperature measurement, history of symptoms, tolerability to the drug, adverse events, and any concomitant therapy. Finger pricks for follow-up blood films and hemoglobin measurements were taken on scheduled days 3, 7, 14, 28, and at any unscheduled visit. Filter papers were collected on day 0 and any day of failure (recurrent fever/parasitemia) for molecular testing. All filter papers were dried and stored in plastic storage bags with desiccant.

### 2.8. Outcomes

Efficacy was assessed by clinical and parasitological outcomes using WHO definitions, with a 28-day follow-up period [34]. As per WHO definitions, early treatment failure (ETF) was defined as development of severe signs or symptoms, or insufficient parasitological response by day three. Patients with *P. falciparum* parasitemia occurring between four and 28 days without fever were classified as late parasitological failure (LPF) and those with fever as late clinical failure (LCF). If no failure was recorded, it was classified as adequate clinical and parasitological response (ACPR).

### 2.9. Molecular Analyses

To distinguish between recrudescence and reinfection, three to four drops of blood were collected on filter paper at day zero prior to treatment, and on any day of recurrent *P. falciparum* parasitemia. DNA was extracted from blood spots dried on filter papers by soaking overnight in 1 mL of 0.5% saponin-1× phosphate buffered saline. The segment was then washed twice in 1 mL of PBS and boiled for 8 min in 100 μL PCR-grade water containing 50 μL 20% chelex suspension (pH 9.5). Recrudescent and new infections were differentiated by typing the highly polymorphic repeat region of MSP2 as previously described [37]. Pre- and post-treatment samples for each patient were compared by running the amplified MSP2 products in adjacent lanes of 1.5% agarose gels. An outcome was defined as recrudescence if at least one identical allele (within 15 bp) was present in both pre- and post-treatment samples. Samples where no alleles matched pre- and post-treatment were classified as new infections [38,39]. If either pre- or post-samples failed to amplify, they were classified as undetermined; all such samples were repeated three times before concluding.

### 2.10. Ethics Statement

The study was approved by the Institutional Review Board of the Faculty of Health Sciences, University of Buea, Cameroon while administrative authorization was obtained from the Southwest Regional Delegation of Public Health. Written informed consent was obtained from the parents/guardians of all children.

## 3. Results and Discussion

### 3.1. Results

#### 3.1.1. Patients

**Figure 1:**
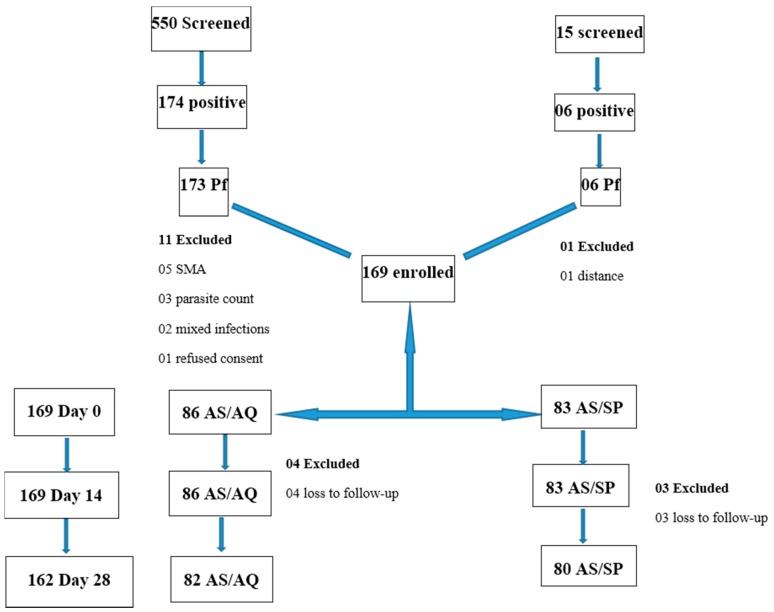
Trial profile. Screening, enrollment, and follow-up of patients.

Of the 565 patients screened from the two study sites, 180 were positive for malaria parasites, of which 179 had *P. falciparum* mono-infection. At the BRH, the slide positivity rate was 40.0% (6/15), of which *P. falciparum* mono-infection accounted for 100% (6/6); whereas, at the Tole health post, the slide positivity rate was 31.6% (174/550) of which *P. falciparum* mono-infection accounted for 99.4% (173/174). In summary, 169 patients were enrolled. The trial profile of the patients and reasons for exclusion are shown in Figure 1. The majority of excluded patients had either severe malaria anemia (Hb < 5 g/dL) or asexual parasitemia below 1000 parasites per microliter. The baseline characteristics of the patients, stratified by drug group, is shown on Table 1.

The mean age, weight, temperature, and hemoglobin level at baseline was similar in patients enrolled in to both treatment group. Furthermore, the geometric mean parasite density and presence of gametocytemia at enrollment was comparable in the AS/SP and AS/AQ groups. Only 9.88% of patients reported using a bed net of any type.

**Table 1:** Baseline characteristics of enrolled patients, stratified by drug group.

Characteristics	Total (*n* = 169) (Range)	Treatment Group (*n*)		*p* Value
		AS/SP (*n* = 83)	AS/AQ (*n* = 86)	
Females (%)	45.6	49.4	41.9	0.325
Mean age ± SD (months)	41.30 ± 23.54 (6–120)	38.23 ± 23.54	44.26 ± 23.30	0.096
Mean weight ± SD (kg)	14.22 ± 4.45 (6–27)	13.61 ± 4.46	14.81 ± 4.39	0.084
Mean temperature ± SD (°C)	38.20 ± 1.35 (36.0–40.9)	38.18 ± 1.42	38.21 ± 1.29	0.891
Mean hemoglobin ± SD (g/dL)	9.94 ± 1.36 (4.33–14.00)	9.89 ± 1.34	10.00 ± 1.39	0.619
GMPD ^&^ (parasites/µL)	11,887 (1000–533,334)	14346	9914	0.101
Gametocytes (%)	19.5	19.3	19.8	0.936
Bed net usage (%)	9.9	10.0	9.8	0.891

^&^ GMPD = Geometric mean parasite density.

#### 3.1.2. Treatment Outcomes

Follow-up was completed for 162 patients to day 28 (Figure 1). The overall treatment failure rate on day 28 was 16.2% (13/80) and 9.8% (8/82) for the AS/SP and AS/AQ groups, respectively. In all, there was only one early treatment failure, 14 (8.6%) late clinical failures and six (3.7%) late parasitological failures, which occurred on day 28 (Table 2). Molecular analysis showed that four sample pairs had bands both for day zero and day 28 (two recrudescence and two reinfections), four samples had bands for day zero only, one sample for day 28, and two sample pairs had no band for both day zero and day 28, classified as undetermined. Seven patients in total, including three and four from the AS/SP and AS/AQ treatment group, respectively, were lost to follow-up.

**Table 2:** Treatment outcomes of AS/SP and AS/AQ after day 28.

Outcome	All Patients (*n* = 169)	Treatment Groups	
		AS/SP (*n* = 83)	AS/AQ (*n* = 86)
No treatment outcome	7	3	4
Loss to follow-up (%)	7 (4.1)	3 (3.6)	4 (4.7)
Early treatment failure (%)	1 (0.6)	0	1 (1.2)
Late clinical failure (%)	14 (8.2)	8 (9.6)	6 (7.0)
Late parasitological failure (%)	6 (3.5)	5 (6.0)	1 (1.2)
Adequate clinical and parasitological response	141 (87.0)	67 (83.8)	74 (90.2)
Infection with different species (%)	0	0	0
New infection *P. falciparum* (%)	2 (1.2)	2 (3.5)	0
Recrudescence (%)	2 (1.2)	2 (3.5)	0
Cure rates-Per Protocol, PCR uncorrected (%, 95% CI)	87.0 (81.8–92.2)	83.8 (75.1–91.5)	90.2 (83.8–96.6)
Cure rates-Per Protocol, PCR corrected (%,95% CI)	88.1 (83.1–93.1)	85.9 (78.2–93.6)	90.2 (83.8–96.6)
Cure rates-Intention-to-treat, PCR uncorrected (%, 95% CI)	83.4 (77.8–89.0)	80.7 (72.2–89.2)	86.0 (78.7–93.3)
Cure rates-Intention-to-treat, PCR corrected (%, 95% CI)	84.4 (78.9–89.9)	82.7 (74.5–90.9)	86.0 (78.7–93.3)

Until day 28, 141 patients from both groups showed an adequate clinical and parasitological response (ACPR). Twenty-one treatment failures were observed during follow-up, constituting one (4.6%), 14 (8.2%), and six (3.5%) ETF, LCF, and LPF, respectively (Table 2). The participant with ETF in the AS/AQ group had an initial parasitemia of 6030/µL but still had parasites together with fever by day three. Per protocol (PP) analysis revealed PCR-corrected cure rates at day 28 of 88.1% overall, 85.9%, and 90.2% from AS/SP and AS/AQ groups, respectively. Uncorrected cure rates at day 28 was 87.0%, 83.8%, and 90.2% from the AS/SP and AS/AQ groups, respectively.

The intention to treat analysis (ITT), uncorrected cure rates at day 28 for AS/SP and AS/AQ were 80.7% and 86.0%, respectively; PCR-corrected cure rates at day 28 for AS/SP and AS/AQ were 82.7% and 86% (Table 2).

#### 3.1.3. Variation of Body Temperature and Fever during Follow-Up

**Figure 2:**
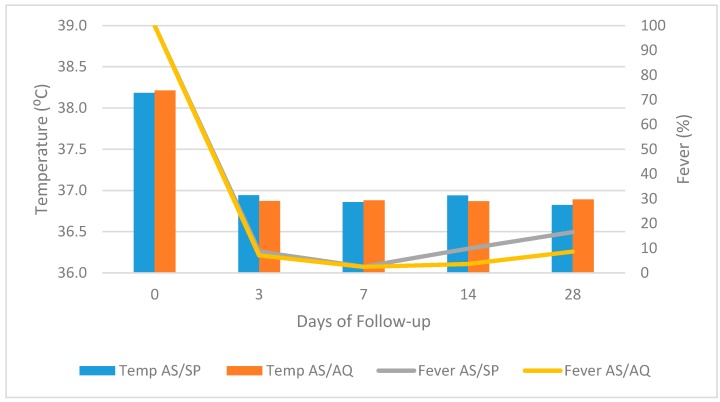
Reduction in mean temperature and proportion of fever cases in the course of treatment.

After initial treatment, the body temperature of all patients went down rapidly and fever clearance at day 3 was 90.9% and 92% in the AS/SP and AS/AQ groups respectively (Figure 2). The mean body temperature at day 3 was significantly lower (*p* < 0.001) than day 0 but similar (*p* > 0.05) to the rest of the follow up days 7, 14 and 28 in both treatment groups.

#### 3.1.4. Evolution in the Percentage of Malaria Parasitemic Cases and Hb Level during Follow-up

**Figure 3:**
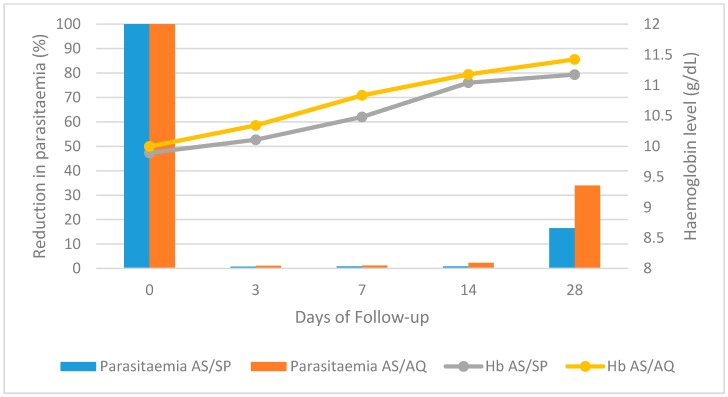
Variation in malaria parasitaemia and haemoglobin level of study participants in the course of treatment.

All patients were positive for malaria parasites at enrolment, with the parasitemia density significantly higher (*p* < 0.001 each) than all other days of follow-up (Figure 3). By day three both treatment regimens proved to be highly efficacious for the treatment of *P. falciparum* malaria as they rapidly cleared parasites and improved hemoglobin level. However, 23 patients still had parasites in their blood by day three, with the malaria parasitemia load similar across days three, seven, and 14, while by day 28, the parasitemia density was significantly higher (*p* < 0.001 each) than the previous three follow-up days.

The mean hemoglobin level of patients in the AS/SP treatment group (9.89 ± 1.34) was similar (*p* = 0.619) to that of the AS/AQ group (10.00 ± 1.39) at baseline. At day 0, 59.1% and 60% of patients were anemic in the AS/SP and AS/AQ groups, respectively. The mean Hb level at day three was similar (*p* = 0.189) to day zero, but increased significantly (*p* < 0.01) in each of the treatment groups during follow up until day 14, whose mean Hb value was similar to that of day 28 (*p* = 0.223) (Figure 3).

#### 3.1.5. Adverse Events

At least one adverse event (AE) was reported in 69.2% (117/169) of patients in both treatment groups during the post-treatment period that was not present on admission. These were possibly or probably related to the study drug and mainly minor or moderate in intensity. The most frequent AEs were cough, dizziness, fatigue, catarrh, and gastrointestinal disorders (nausea, abdominal pain, and diarrhea) (Table 3). A total of 36 (43.4%) and 81 (94.2%) patients in the AS/SP and AS/AQ group, respectively, experienced AEs by day three. By day seven, the number of patients with AEs had reduced to two (4.5%) and eight (17.7%) in the AS/SP and AS/AQ groups, respectively.

**Table 3:** Frequency of mild-to-moderate adverse events on days 3–7 (related or not to treatment) with artesunate-fansidar (AS/SP) in comparison with artesunate-amodiaquine (AS/AQ).

Event	AS/SP (*n* = 83)	AS/AQ (*n* = 86)
At least one adverse event	36	81
Abdominal pain	0	2
Cough	16	14
Catarrh	6	4
Diarrhoea	0	1
Dizziness	6	28
Anorexia	0	3
Fatigue	6	22
Nausea	2	4
Jaundice	0	3

### 3.2. Discussion

Global efforts to eradicate malaria are being threatened on an unprecedented scale by rapidly growing resistance of *P. falciparum* to conventional antimalarial drugs [40]. Early diagnosis and prompt treatment remain the mainstay for the control of the disease [41], with routine monitoring of the efficacy of antimalarials necessary to identify and contain the spread of resistant parasites.

This comparative evaluation of the efficacy of AS/SP and AS/AQ shows that both ACTs remain effective in the treatment of malaria due to *P. falciparum* in Cameroon. The ACPR recorded at day 28 in the AS/SP (83.8%) and AS/AQ (90.2%) groups is in line with a previous study conducted at four different urban centers in Cameroon, where ACPR at day 28 of 88.5% and 88.3% were reported in the AS/SP and AS/AQ groups, respectively [30]. Both combinations demonstrate good activity against the asexual forms of the parasite and, thus, remain effective in the treatment of malaria in Cameroon. High therapeutic efficacies of ACTs have also been reported in Cameroon [28,42,43] and other African countries (Kenya, Mali, and Congo) with different levels of endemicity with noted chloroquine resistance [41,44,45].

Nevertheless, ACPR >90% at day 28 for both combinations have been reported in Sudan and the Gambia [46] and in a recent meta-analysis of individual patient data for AS/AQ by WWARN [47]. The slightly lower efficacies recorded in this study may accrue to the depreciation of both SP and AQ as monotherapies since complete clearance of parasites depend on the level of efficacy of the partner drug [2]. SP and AQ have been largely used in Cameroon as monotherapies and are still available in the saturated pool of antimalarials in the country [27]. Unlike in other countries and areas where some monotherapies remain highly effective in the treatment of uncomplicated malaria, both partner drugs, may have been compromised by resistance, reportedly at 46.6% and 18% for SP and AQ, respectively, in the region [48].

The PCR-corrected cure rates, indicative of the true drug efficacy, suggests that AS/AQ (90.2%) was a slightly better treatment than AS/SP (85.9%). Furthermore, both new infections and recrudescence occurred only in the AS/SP group at day 28. The decreased efficacy of AS/SP compared to AS/AQ probably reflects the decreased sensitivity to SP *vis-à-vis* AQ in the region [48]. However the observed failure rate of 6.0% in the AS/SP group confirms that findings that the addition of artemisinin derivatives to standard antimalarials greatly improves treatment success of SP as monotherapy [2].

Artemisinin derivatives remain the most potent and rapidly acting schizonticidal drugs with limited resistance. The addition of artesunate to previously standard monotherapeutic antimalarial regimens reduces treatment failure and recrudescence, thus slowing down the development of resistance. Thus, the probability that an infected patient will have parasite resistant to both drugs and/or carry gametocytes is greatly reduced [49]. In fact only 12% of cases had gametocytemia after 14 days in this study, in contrast to the reported 68% and 28% post-treatment gametocytemia in patients under SP and AQ therapies, respectively [43]. Therefore, the ACTs tested in this study were effective in reducing gametocytemia, decreasing the number of patients with gametocytes during follow-up, gradually, until day 14. According to [2], artemisinin appears to exert its gametocidal effect by preventing the development of new gametocytes rather than clearing existing ones. This ability of artesunate to reduce post treatment gametocytemia is important since the sexual stages of the parasite are important for the transmission of malaria via mosquito vectors and hence might reduce transmission. 

Our results show an amelioration of Hb levels with duration of treatment of patients in both drug groups. This is important as a rise in Hb values and a reduction of the proportion of anemic children supports the fact that malaria parasites were effectively cleared from the blood and red cells count rose after treatment. Other studies in Buea and in Sudan reported similar increase in Hb values following treatment with ACTs [43]. This is an important observation since anemia associated with malaria is a major cause of morbidity and mortality in malaria endemic area, especially in young children and pregnant women [50]. 

Adverse events were reported in both treatment groups, with dizziness and fatigue being the most frequent. Nevertheless, more AEs occurred in the AS/AQ compared to the AS/SP group, due perhaps to the amodiaquine component that has been known to cause serious side effects such as hepatitis, decrease mean absolute neutrophil count and induced neutropenia [51]. Moreover, AEs due to AQ may be associated with genetic polymorphisms in the CYP450 genes that have been shown to reduce CYPCC8 expression in sub-Saharan Africa, leading to the poor metabolizer phenotype [52,53]. Although most of the AEs were self-limiting and disappeared by day 14, further evaluation of the safety of the AS/AQ combination is necessary, since its undesirable side effects remain a problem to indigenous of malaria endemic countries.

This study is limited by the fact that it was conducted since 2006, over nine years ago with possibly genetically different parasites due to ecological changes that affect vector populations and transmission dynamics. Nevertheless, it provides invaluable baseline information, especially as no reports of the efficacy of these ACTs exist in the region, considered to be a hotspot where resistance to chloroquine was first reported in Cameroon [25]. The delay in publication accrues mainly to the fact that molecular analyses to distinguish recrudescence from reinfection was not possible until recently.

Secondly, there was no upper limit of inclusion parasitemia, although patients with high initial parasitemia have reported to be at higher risk of recrudescence [54]. Nonetheless, the drug was effective in treating the patient with the highest load of 533,334 asexual parasites per microliter.

## 4. Conclusions

The two artemisinin-based combination therapies tested (AS/AQ and AS/SP were efficacious and well-tolerated in the treatment of uncomplicated malaria around the slope of Mount Cameroon. Although both combinations are very good in reducing the prevalence of anemia and gametocytemia, AS/AQ was slightly more effective than AS/SP, while AS/SP appeared better tolerated.
